# A Manual of Veterinary Therapeutics

**Published:** 1895-02

**Authors:** 


### REVIEWS.

*A Manual of Veterinary Therapeutics.* BY E WALLIS HOARE, F.R.C.V.S. English veterinary literature has lacked for many years the existence of a modern, up-to-date work on veterinary therapeutics. While every English-speaking veterinarian feels a debt of gratitude to Finlay Dunn for his excellent work, yet so much has been recently added to the knowledge of veterinary medicine and therapeutics that a work just issued from the pen of the above author and controlled in the United States by the well-known veterinary publishing house of W. R. Jenkins, New York, will more than fill a long-felt want. The plan adopted in considering drugs and their actions and uses will be fully appreciated by every veterinarian. The concise, clear, and definite manner in which the whole subject is considered will find rapidly for it a place in the hands of every busy, progressive practitioner in North America. Surely one can well say that the growth in English veterinary literature has been well augmented by this addition, and the path of every student of veterinary medicine will be made lighter and easier to travel through a perusal of its well-filled pages.

